# Quantification of Blumenol Derivatives as Leaf Biomarkers for Plant-AMF Association

**DOI:** 10.21769/BioProtoc.3301

**Published:** 2019-07-20

**Authors:** Elisabeth Mindt, Ming Wang, Martin Schäfer, Rayko Halitschke, Ian T. Baldwin

**Affiliations:** 1 Department of Molecular Ecology, Max Planck Institute for Chemical Ecology, Jena, Germany

**Keywords:** Arbuscular mycorrhizal fungi, AMF, Fungus-plant symbiosis, Leaf biomarker, Shoot biomarker, Blumenol derivatives, UHPLC-MS/MS technique

## Abstract

Symbiotic interactions between arbuscular mycorrhizal fungi (AMF) and plants are widespread among land plants and can be beneficial for both partners. The plant is provided with mineral nutrients such as nitrogen and phosphorous, whereas it provides carbon resources for the fungus in return. Due to the large economic and environmental impact, efficient characterization methods are required to monitor and quantify plant-AMF colonization. Existing methods, based on destructive sampling and elaborate root tissue analysis, are of limited value for high-throughput (HTP) screening. Here we describe a detailed protocol for the HTP quantification of blumenol derivatives in leaves by a simple extraction procedure and sensitive liquid chromatography mass spectrometry (LC/MS) analysis as accurate proxies of root AMF-associations in both model plants and economically relevant crops.

## Background

The widespread mutualistic relationship between AMF and plants involves not only the beneficial exchange of nutrients between the involved partners; phosphorous and nitrogen are supplied by the fungus and carbon is supplied by the plant in exchange; but is also thought to regulate plant growth and tolerance to various biotic and abiotic stresses (Maier *et al.*, 1995; Barin *et al.*, 2013; Aliferis *et al.*, 2015; Wang *et al.*, 2018). These interactions have fueled vast research programs and, in conjunction with dwindling natural phosphorous supplies, are of high interest for sustainable agriculture (Basu *et al.*, 2018). Until now, the available approaches to measure and quantify AMF-plant associations require excavation of the roots followed by microscopic analysis, transcript analysis or quantification of fungal fatty acids (Barin *et al.*, 2013). However, these methods are impractical for HTP screening due to the damage that results from root sampling, as well as being laborious (Barin *et al.*, 2013; Wang *et al.*, 2018). Hence, an HTP screening technique is needed to empower research and development in breeding programs for improved AMF-plant associations. Even though AMF colonization leads to systemic responses throughout the plant, until recently no AMF-specific metabolic response has been detected in plant parts other than in roots (Aliferis *et al.*, 2015; Hill *et al.*, 2018). The described protocol is based on a MeOH extraction of leaf tissue followed by Ultra High Performance Liquid Chromatography Mass Spectrometry (UHPLC-MS) analysis as described byWang *et al.* (2018). The concentrations of foliar 11-hydroxy- and 11-carboxyblumenol C derivatives are not detectable in non-mycorrhized plants and are positively and quantitatively correlated with AMF root colonization and are transported from roots to the leaves after the formation of root-AMF associations (Wang *et al.*, 2018). This protocol facilitates an HTP, non-destructive and quantitative characterization of AMF associations in various model and agricultural crop plant species.

## Materials and Reagents

1. Pipette tips
2. 96-well microplates with full skirt (Sapphire, Greiner Bio-One, catalog number: 652270)
3. (Optional) Individual tubes
*
Note: Individual tubes can be used instead of 96-well BioTubes^TM^ for small batches of samples.
*
  - 2 ml Eppendorf Safe-Lock tubes (Eppendorf, catalog number: 0030120094)
  - 1.5 ml screw neck vials N9 (Macherey-Nagel, catalog number: 702282)
  - N9 PP screw caps (Macherey-Nagel, catalog number: 702287.1)
  - Steel balls Ø 4 mm (ASKUBAL, G100-1.4034, catalog number: 503012)
4. Sealing film for 96-well microplates (Zone-free^TM^, EXCEL Scientific, catalog number: ZAF-PE-50)
5. 96-well PCR Plate (µltraAmp, Sorenson^TM^ BioScience Inc, catalog number: 21970)
6. Domed 8-strip PCR caps (Eppendorf, catalog number: 0030124839)
7. Steel balls Ø 3 mm (ASKUBAL, G100-1.4034, catalog number: 505001)
8. Leaf material
9. Liquid nitrogen
10. MilliQ water
11. Deuterated internal standard: D_6_-ABA (HPC Standards GmbH, 10 µg ml^-1^ in MeOH)
12. Acetonitrile (VWR International, HiPerSolv CHROMANORM^®^ for LC-MS, catalog number: BDH83640.100E)
13. Formic acid (Fluka, for mass spectrometry, catalog number: 94318)
14. Methanol (Merck, Gradient grade for LC LiChrosolv^®^, catalog number: 1060072500)
15. Roseoside (Wuhan ChemFaces Biochemical Co., Ltd., catalog number: CFN98916)
16. Corchoionoside C (Wuhan ChemFaces Biochemical Co., Ltd., catalog number: CFN99859)
17. Blumenol C glucoside (Wuhan ChemFaces Biochemical Co., Ltd., catalog number: CFN99424)
18. Byzantionoside B (Wuhan ChemFaces Biochemical Co., Ltd., catalog number: CFN99871)
19. Extraction buffer with deuterated internal standard D_6_-ABA (see Recipes)

## Equipment

1. Stainless steel spatula
2. Stainless steel tweezers
3. 96-well tube racks (BioTube^TM^, Simport^®^ Scientific, catalog number: T101-1 and T100-20)
4. Sealing mats for 96-well tube racks (ArctiSeal^TM^, Arctic White LLC, catalog number: AWSM-2002RB)
5. Cooling containers (Heathrow Scientific, True North^®^)
6. Centrifuge (Eppendorf, model: 5415 R)
7. Multipipette (Multipette^®^ Xstream, Eppendorf, catalog number: 4986000025)
8. Mortar and pestle (Haldenwanger^TM^, Fisher Scientific)
9. Analytical balance (Sartorius, model: BP121S)
10. 8-channel electronic pipette (Eppendorf, Xplorer^®^, 50-1,200 µl, catalog number: 4861000163)
11. Tissue homogenizer (Geno/Grinder^®^ 2000, SPEX SamplePrep)
12. Cooled centrifuge equipped with 96-well plate rotor (Eppendorf, model: 5804 R, rotor A-2-DWP)
13. UHPLC triple quadrupole MS instrument [Ultimate 3000 RSLC (Thermo Fisher Scientific); EVO-Q Elite^TM^ (Bruker)]
14. UHPLC column (ZORBAX Eclipse XDB-C18, 50 x 3.0 mm, 1.8 µm, Agilent, catalog number: 981757-302)
-80 °C freezer

## Software

1. MS Data Review Version 8.2.1 (MS Workstation, Bruker Daltonics)

## Procedure

*Notes:*

- *In order to test the applicability of the method for the analyzed plant/AMF species, it is advised to perform an initial test screen with root tissue as the abundance of blumenol derivatives in root tissue is higher than in leaves.*
- *
Blumenol levels can vary in different shoot tissues (Wang et al., 2018). Harvesting tissue samples from leaves at comparable developmental stages will reduce variation and allow better comparisons between plants.
*
- *
Blumenol levels reliably indicate AMF colonization 3 weeks after inoculation (Wang et al., 2018).
*
  - Collection and preparation of leaf material
    1. Harvest leaves and immediately freeze in liquid nitrogen using stainless steel tweezers. Store at -80 °C until processing the samples.
    2. Grind the frozen leaf material with mortar and pestle under liquid nitrogen.
    3. Aliquot approximately 100 mg leaf material with a pre-cooled stainless steel spatula into liquid nitrogen-precooled and pre-weighted 96-well BioTube^TM^ racks containing two steel balls (Ø 3 mm). Record the exact mass and leave the samples on liquid nitrogen for extraction or store at -80 °C.
*
Note: Instead of 96-well BioTubes^TM^, 2 ml Eppendorf tubes equipped with two steel balls (Ø 4 mm) can be utilized.
*
  - Extraction
    1. Add 800 µl of ice-cold extraction buffer containing the internal standard D_6_-ABA to each tube with an 8-channel pipette. Replace the tube caps with a rubber sealing mat.
*Note: Samples should be kept on ice during the extraction procedure.*
    2. Homogenize the samples in a Geno/Grinder^®^ for 60 s at 1,000 strokes per minute (Geno/Grinder^®^ 2000 setting: 1x at 000).
    3. Centrifuge the samples at 2,000 *× g* for 20 min at 4 °C, transfer the supernatant to a new 96-well BioTube^TM^ rack or Eppendorf tubes without steel balls and centrifuge again under the same conditions.
    4. Transfer 100 µl of the supernatant into skirted 96-well microplates and close wells with sealing film for LC-MS/MS analysis.
    5. As the sealing film is not suitable for long-term freezer storage, transfer 190 µl of the supernatant into 96-well PCR plates and seal with 8-strip caps as freezer backup.
*Note: In case Eppendorf tubes are used, transfer 700 µl of the supernatant to 1.5 ml screw neck vials (vials are stored in the freezer for re-analysis).*
    6. Prepare a mixed quality control (QC) sample for each 96-well plate by combining 10 µl aliquots of each sample of the plate in a 1.5 ml screw neck vial.
    7. Use the extraction buffer as blank and for signal background calculations.
  - UHPLC-MS/MS

For the chromatographic separation, utilize an Agilent ZORBAX Eclipse XDB-C18 column. The mobile phase consists of 0.1% (v/v) acetonitrile and 0.05% (v/v) formic acid in MilliQ H_2_O for solvent A and 100% methanol as solvent B. The mobile phase gradient of the UHPLC method is shown in Table 1. The UHPLC instrument parameters comprise:

**Table:** 

Flow rate	0.5 ml min^-1^
Sample tray temperature	10 °C
Sample injection volume	5 µl
Column temperature	42 °C

**Table 1.** Mobile phase gradient of the UHPLC run

Time [min]	% mobile phase A	% mobile phase B
0.0-1.0	90	10
1.0-1.2	90-65	10-35
1.2-3.0	65-58	35-42
3.0-3.4	58-0	42-100
3.4-4.4	0	100
4.4-4.5	0-90	100-10
4.5-5.5	90	10

The Bruker EVO-Q Elite^TM^ triple quadrupole MS system is used in multiple reaction monitoring (MRM) mode. The heated electrospray ionization (HESI) source settings consist of:

**Table:** 

HESI spray voltage	± 4,500 V
Cone temperature	350 °C
Probe temperature	300 °C
Cone gas flow	35
Nebulizer gas flow	60
Probe gas flow	55

 System performance and general ESI parameters can be evaluated by injecting a standard solution of related blumenol glycoside compounds: Roseoside (Wuhan ChemFaces Biochemical Co., Ltd.; catalog number: CFN98916), Corchoionoside C (CFN99859), Blumenol C glucoside (CFN99424), Byzantionoside B (CFN99871). Standards for the 11-hydroxy- and 11-carboxyblumenol C derivatives are not commercially available.

The MRM settings for the detection of specific blumenol derivatives are shown in Table 2 and a recording window of 1 min is set at the expected retention time (R_T_). The displayed compound table has been tested and found to be widely applicable. Additional markers can be identified in order to extend the method beyond the current list of plant species that have been investigated (Wang *et al.*, 2018):

**Table:** 

Barley	*Hordeum vulgare*
Barrel clover	*Medicago truncatula*
Common rice	O*ryza sativa*
Common wheat	*Triticum aestivum*
Potato	*Solanum tuberosum*
Stiff brome	*Brachypodium distachyon*
Tomato	*Solanum lycopersicum*
Wild tobacco	*Nicotiana attenuata*

**Table 2.** Quantifier and Qualifier m/z fragments used to detect blumenol derivatives in plant leaves

Compound name	R_T_ [min]	Precursor ion m/z^§^	Quantifier m/z [collision energy]	Qualifiers m/z [collision energy]
11-hydroxyblumenol C-Glc*	2.78	(+) 389.2	209.2 [7.5 V]	227.2 [2.5 V], 191.1 [12.5 V], 163.1 [15.0 V], 149.1 [17.5 V]
11-hydroxyblumenol C-Glc-Glc*	2.47	(+) 551.3	209.2 [10.0 V]	389.2 [2.5 V], 227.2 [7.5 V], 191.1 [15.0 V], 149.1 [20.0 V]
11-carboxyblumenol C-Glc*	3.17	(+) 403.2	195.1 [12.5 V]	241.2 [2.5 V], 223.2 [7.5 V], 177.1 [15.0 V]
	3.17^&^	(+) 241.2	195.1 [10.0 V]	223.2 [5.0 V], 177.1 [15.0 V]
11-carboxyblumenol C-Glc-Glc†	3.10	(+) 565.2	195.1 [15.0 V]	403.2 [2.5 V], 241.2 [4.5 V], 223.2 [15.0 V]
11-carboxyblumenol C-Mal-Glc†	3.60	(+) 489.2	195.1 [12.5 V]	241.2 [2.5 V], 223.2 [7.5 V], 177.1 [15.0 V]
abscisic acid (ABA)^**,^	4.00	(-) 263.2	153.0 [9.0 V]	
blumenol A-Glc^**,^	2.46	(+) 387.2	207.1 [8.0 V]	225.2 [5.0 V], 149.1 [18.0 V], 135.1 [16.0 V], 123.1 [23.0 V]
	2.46	(-) 385.2	153.1 [14.0 V]	
D_6_-ABA^¶^	4.01	(-) 269.2	159.0 [10.0 V]	

^§^ Ionization polarity is indicated in parentheses. * Verified by NMR. ** Verified with authentic standard. ^&^ The fragmentation of the m/z 241.2 aglycon precursor [M+H-Glc]^+^ allows for a sensitive MRM detection in addition to the MRM of the m/z 403.2 molecular ion [M+H]^+^. ^ǂ^ Blumenol A and abscisic acid are not induced by AMF (Wang *et al.*, 2018) and can be used as internal standards to evaluate the overall functionality of the carotenoid biosynthesis in the analyzed plant as well as providing valuable information about instrument performance. † The identity of 11-carboxyblumenol C-Glc-Glc and 11-carboxyblumenol C-Mal-Glc detected in rice has not been confirmed. ^¶^ Internal Standard (typically showing 20-30% relative standard deviation after full extraction/analysis procedure).

The prepared QC samples will be analyzed repeatedly after every 15 to 20 samples with the identical UHPLC-MS/MS method. Comparisons of the QC runs will allow monitoring instrument performance and detecting retention time shifts or changes in mass spectrometer sensitivity in larger sample batches.

## Data analysis

**Figure 1.**
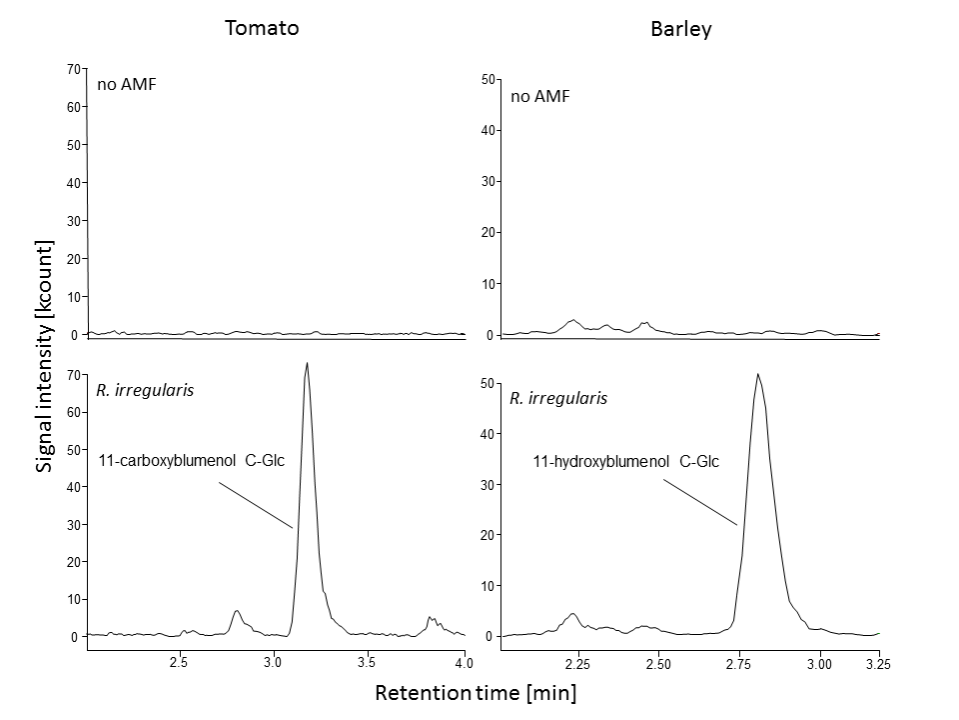
Chromatographic output for blumenol derivatives in different crop plants. Blumenol derivatives were extracted from leaf tissue of control plants (no AMF) and plants inoculated with *Rhizophagus irregularis*. 11-carboxy- and 11-hydroxyblumenol C-Glc were detected in AMF-colonized tomato (*Solanum lycopersicum*) and barley (*Hordeum vulgare*) plants, respectively. Details of the inoculation procedure can be found inWang *et al.* (2018).

EXAMPLES of the blumenol derivative signals detected in leaves of barley (*Hordeum vulgare*) and tomato (*Solanum lycopersicum*) plants with and without AMF colonization are shown in Figure 1.

Peak area integration for the targeted compounds and the internal standard is carried out via the software MS Data Review Version 8.2.1 (MS Workstation, Bruker Daltonics). The analyte peak area is normalized to the internal standard D_6_-ABA and concentrations of blumenol derivatives are calculated as D_6_-ABA equivalents (ng mg^-1^ fresh mass) using the following equation:

$$\frac{\left(\frac{{PA}_{\;analyte}}{{PA}_{D_{6}-ABA}\;}\;x\;\;{{mass}_{D}}_{6-\;ABA}\right)}{{mass}_{sample}\;}$$

${PA}_{analyte}\;$and ${PA}_{D_{6-ABA}}$ represent the peak areas (in counts) of the target analyte and internal standard, respectively.

${mass}_{D_{6-ABA}\;}\;$is the amount of internal standard (in ng) that is introduced to the sample via the extraction buffer.

${mass}_{sample}\;\;$corresponds to the fresh mass (in mg) of the leaf tissue sample.

## Recipes

1. Extraction buffer including internal standard D_6_-ABA
200 ml MilliQ H_2_O
  800 ml MeOH (gradient grade for LC)
1.25 ml of 10 ng µl^-1^ D_6_-ABA (final concentration of 10 ng per 800 µl extraction buffer)
